# New products

**DOI:** 10.1155/S1463924695000356

**Published:** 1995


Journal of Automatic Chemistry, Vol. 17, No. 6 (November–December 1995), pp. 225–227

### New products

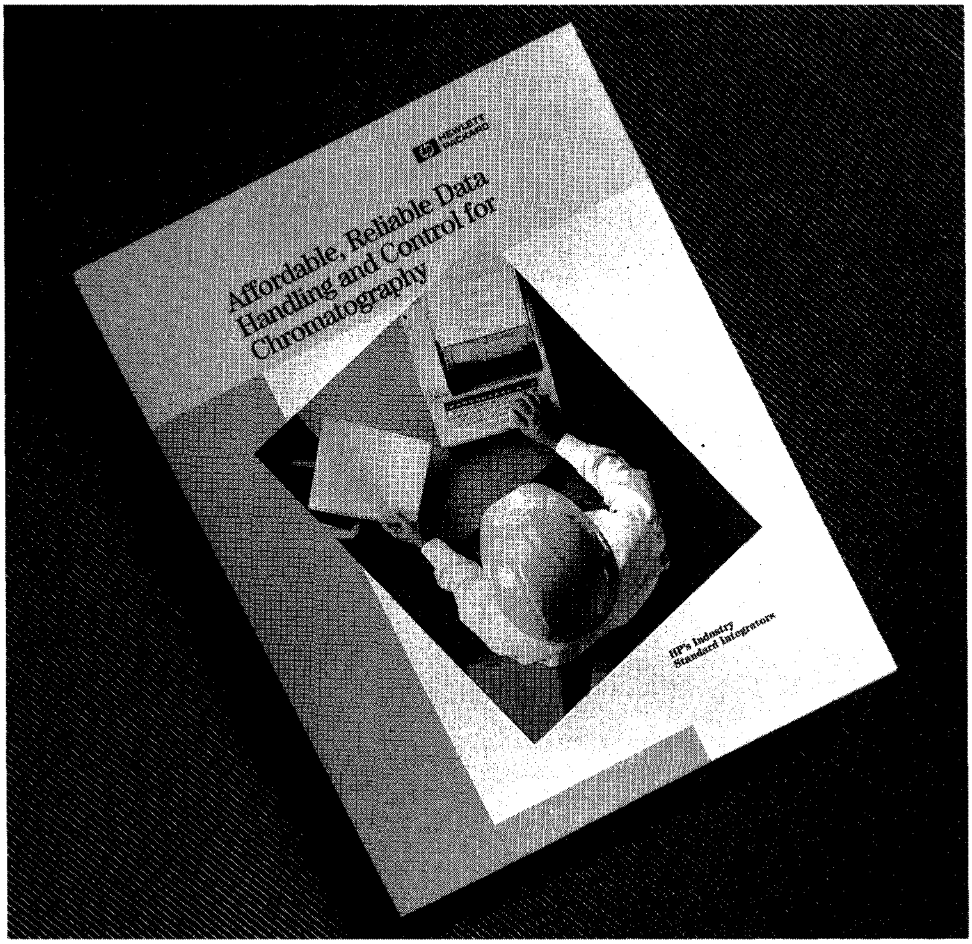

*Figure 1. Hewlett-Packard S.A. has published a brochure (Literature 5963-5083E) that describes affordable, entry-level data-handling and analytical instrument-control solutions for chromatography. The brochure is for laboratory managers, chemists, analysts and purchasing agents who need robust, low-cost and reliable data handling and control for HP and non-HP analytical instruments. The brochure discusses the data-handling and instrument-control features of the HP 3396 Series III and 3395 Series III integrators. It includes details on how software applications can customize the HP 3396 Series III integrator for use with the HP 6890 series gas chromatograph or the HP 1050 series high-performance liquid chromatograph. Details also are included on how the HP 3395 Series III integrator can be used with non-HP instruments.*

*The brochure is available without charge from any HP sales office. Or contact Hewlett-Packard, Enquiry Fulfilment Department, PO Box 533, 2130 AM Hoofddorp, The Netherlands.*

### Exporter of the Year

Sirius Analytical Instruments were the recent recipients of an *Exporter of the Year* award. Sirius manufactures an instrument which investigates how drugs ionize and partition across membranes. It is sold largely to pharmaceutical companies and while first sales were in the UK, in the last two years, the sales activity has been almost exclusively by export. Sales in certain countries are direct and in others, particularly in Southern Europe and USA, and Scandinavia are by distributors. The company's staff includes chemists who are acknowledged experts in the field. Many Sirius authorized papers have appeared in international journals and marketing activity is often focused on the presentation of material at international seminars.

*Details from Sirius Analytical Instruments, Riverside, Forest Row Business Park, Forest Row, East Sussex RH18 5DW, UK. Tel.: 0134 282 4036. E mail: Sirius@cix.compulink.co.uk.*

### FT-NIR analyser

A new addition to the Genesis FTIR Series of instruments, Genesis NIR covers the wavelength range from 10 000 to 2000 wavenumbers with a resolution of 1 $cm^{-1}$.

The NIR Genesis Series spectrometers bring the fundamental advantages of Fourier Transform Infrared spectroscopy to the near infrared spectral region. Excellent wavelength precision stems from the internal HeNe laser used to trigger data acquisition. The wavelength precision of 0·05 $cm^{-1}$ is crucial for maintaining the precision and accuracy of PLS Quant and Discriminant analysis methods commonly used for sample evaluation. Throughput and multiplex advantages (Jacquinot and Felget advantages) deliver faster analysis with better spectral quality for remote sampling with fibre optic accessories.

Genesis NIR spectrometers include a full-size sample compartment for transmission sample analysis. Popular reflectance accessories are available on pre-aligned, pinned baseplates for diffuse and specular reflectance analysis. An external beam, controlled by the computer, accesses fibre optic sampling probes for remove analysis. The main sample compartment remains free for other analysis methods.

*For further information contact Paul Carter, ATI Unicam, York Street, Cambridge CB1 2PX, UK. Tel: 01223 358866, fax: 01223 312764.*

### Clenbuterol and CAMAG

Clenbuterol is illegally used as a growth accelerating drug in the production of livestock, particularly beef and fattening calves. It is also used as a sedative during transport of cattle. The Spanish Organizacion de Usarios y Consumidores (OCU), a member of the International Organization of Consumer Associations, has reported that in a test of 936 samples of calves' liver from 12 EU countries, 10% were found to contain clenbuterol (see figure 2).

CAMAG offers a procedure for the identification and determination of clenbuterol in meat, animal feed and urine of livestock, which is based on modern thin-layer chromatography. After sample clean-up the extract is chromatographed on silica, then derivatized with Bratton-Marshall reagent. For qualitative identification, the plate is visually evaluated. Samples with suspected clenbuterol content are chromatographed on another plate, derivatized and scanned densitometrically by absorbance at 525 nm. The detection limit was determined at 5 ppb in meat and in animal feed and at 2 ppb in urine. Advantages of using this method include positive identification in biological samples; quantitative determination; and high sample throughput at low operating costs.

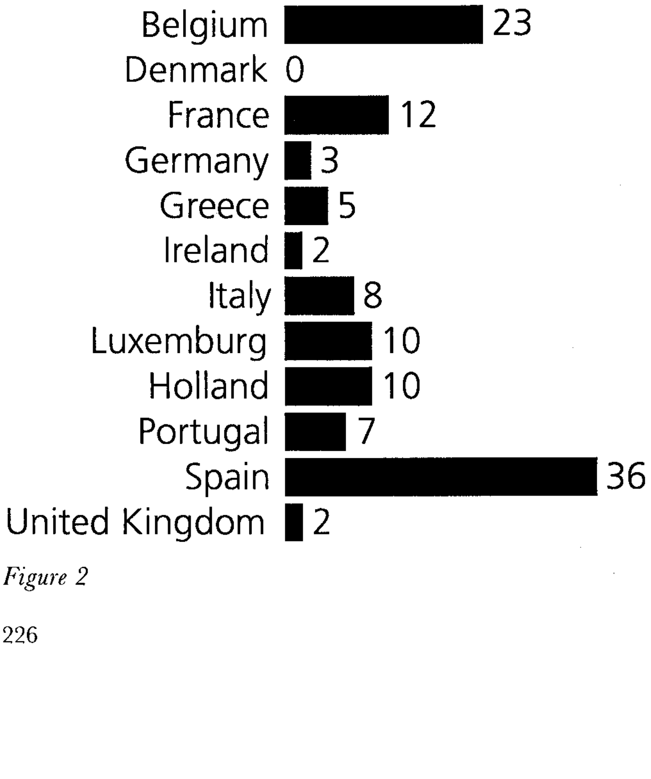

*Figure 2*

*Details from CAMAG Chemie-Erzeugnisse und Adsorptionstechnik, AG, Sonnenmattstrasse 11, CH-4132 Muttenz 1, Switzerland. Tel.: 061 467 34 34; fax: 061 461 07 02.*

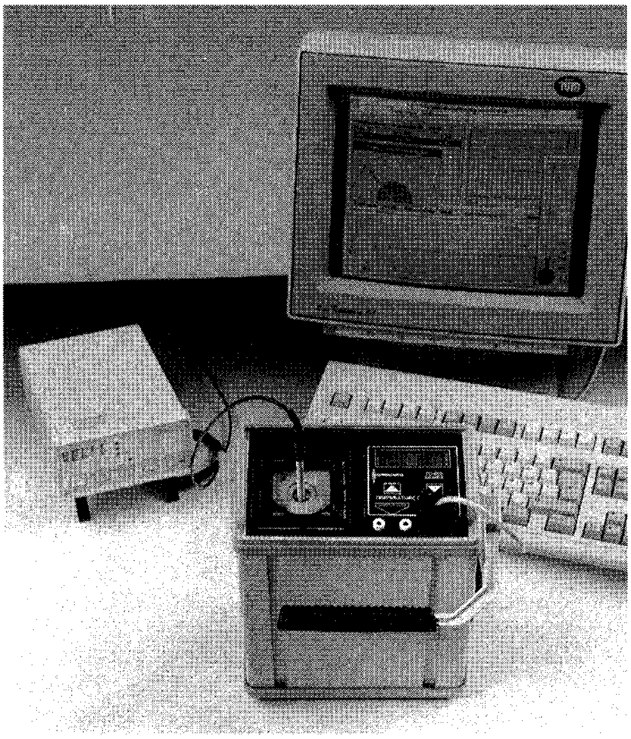

*Figure 3. A complete system for fully automatic temperature calibration is being offered by Tek Know. The system consists of a temperature calibrator (furnace) which generates a stable and accurate temperature reference, and Tek Know's SM 300 signal conditioner which will accept inputs from most thermocouples and RTDs. Windows based software presents calibration results either in table, certificate or graphic form. Further information from Tek Know, Vesterbrogade 149, 1620 Copenhagen V, Denmark. Tel.: 45 33 27 03 01; fax: 45 33 27 03 00.*

### Quattro II

A brochure about the VG Quattro II is now available from Fisons Instruments/VG Organic. The Quattro II was designed to offer the highest performance and sensitivity in all modes of MS and MS/MS analysis. It is adaptable to changing analysis requirements. Features include a flexible multiple inlet system, GC particle beam, LSIMS, APcI and electrospray, plus full control of a range of HPLC and GC systems and autosamplers. Its field-upgradeable design allows the user the freedom to use all of today's advanced inlet techniques and the potential to access future developments. A comprehensive range of inlets ensures that organic compounds of widely differing mass, polarity and structure can be analysed.

Quattro II was designed to integrate a range of GC systems, including the HP 5890 and the CE Instruments 8000 series gas chromatographs featuring the option of Digital Pressure/Flow Control (DPFC). Optimized for high resolution chromatography, the 8000 series GCs support a flexible range of advanced injectors including variable geometry split/splitless and true cold on-column injectors. The system's rugged API (Atmospheric Pressure Ionization) LC/MS interface delivers the highest sensitivity for the analysis of labile compounds, and both atmospheric pressure chemical ionization (APcl) and megaflow electrospray inlets are available. Switching between the two ionization modes is simple and can be accomplished in minutes without venting the system.

*For more information contact Fisons Instruments, Queens Avenue, Macclesfield, Cheshire, SK10 2BN, UK. Tel.: 01625 434343; Fax: 01625 434335.*

### Analytical balances to fit reduced budgets

Mettler-Toledo AG has developed a new series of analytical balances for day-to-day laboratory needs. The AB54 measures up to 51 g with a 0·1 mg display; the AB154 measures up to 101 g; and the AB204 measures up to 210 g. Operator guidance is simple and logically structured, so even temporary staff can perform accurate measurements effortlessly. An adjustable vibration adapter, means that this measurement performance is also possible in an unfavourable operating environment. A test weight is supplied for periodic and rapid adjustment of the balance, for example, to ensure compliance with in-house ISO standards. A rechargeable PowerPack is supplied which enhances flexibility. Density kits are available which allow determination of the densities of solids and liquids. An optional LocalCAN universal interface ensures communication between balance and peripherals (printers, auxiliary displays, foot switches and via RS232C to all computers).

*Details from Mettler-Toledo AG, CH-8606 Greifensee, Switzerland. Tel.: 1 944 22 11; fax: 1 944 30 90.*

### Mathcad

McGraw-Hill Book Company Europe are now international distributors of Mathcad 6.0 Student Edition technical calculation and communications software, the 'Explorations with Mathcad' series of electronic books, and the Mathcad User's Guide printed book.

Mathcad 6·0 Student Edition is the first product to integrate World Wide Web (WWW) connectivity, messaging and powerful maths, science and engineering calculation tools in an easy-to-use environment. It is intended for students and teachers in college and university courses, who need to take maths as part of their required syllabus. Mathcad allows users to enter formulae and equations in live maths notation, create live graphs and animation, share and access information via e-mail and the WWW, and produce presentation quality output. Users can also participate in the Mathsoft Back-to-School Puzzle Competition, a contest in which students from around the world can test their maths problem-solving skills and compete for prizes including a Pentium computer, air travel awards and cash.

The 'Exploration with Mathcad' books incorporate MathSoft's Mathcad Engine technology so they can be used independently of Mathcad while retaining its intuitive 'live maths' environment.

Mathcad is currently available for PC, Macintosh and UNIX workstations; and in German, French, Spanish and Japanese.

*For more information contact David Crowther, Marketing Manager, McGraw-Hill Book Company Europe Ltd, Shoppenhangers Road, Maidenhead, Berkshire, SL6 2QL. Tel.: 01628 23432; fax: 01628 770224.*

### Microsampling

Chromacol has developed a new type of glass support sleeve allowing the use of 200 and 300 µl microsample vials with a wide range of autosamplers which would normally operate with the larger 12 × 32 mm, 2 ml vials. These new glass sleeves, SV-S1·1G, are compatible with HP 7673A autosamplers; transparent so the sample and needle can be seen; and they include a ceramic write on label for GLP and traceability.

These new sleeves are available packed in a blue anodised, indexed rack, holding 25 vials, the same number of vials in each segment of the HP 7673A autosampler.

*For further information contact Jane Willacy at: Chromacol Ltd, Glen Ross House, Unit 3, Mundells Industrial Centre, Welwyn Garden City, Hertfordshire, AL7 1E, UK. Tel.: 01707 394949; fax: 01707 391311.*

